# Empanelment: A foundational component of primary health care

**DOI:** 10.12688/gatesopenres.13059.1

**Published:** 2019-10-31

**Authors:** Trudy Bearden, Hannah L. Ratcliffe, Jonathan R. Sugarman, Asaf Bitton, Leonard Abbam Anaman, Gilbert Buckle, Momodou Cham, Diane Chong Woei Quan, Fatanah Ismail, Badarch Jargalsaikhan, Wujung Lim, Nik Mazlina Mohammad, Isaac C.N. Morrison, Bolormaa Norov, Juhwan Oh, Gandiimaa Riimaadai, Sondi Sararaks, Lisa R. Hirschhorn

**Affiliations:** 1 Comagine Health, Seattle, WA, 98133, USA; 2 Ariadne Labs, Brigham & Women’s Hospital and Harvard T.H. Chan School of Public Health, Boston, MA, 02215, USA; 3 Ghana Health Service Headquarters, Accra, Ghana; 4 Independent Consultant, Accra, Ghana; 5 Christian Health Association of Ghana, Accra, Ghana; 6 Ministry of Health, Putrajaya, Malaysia; 7 Mongolian National University of Medical Sciences, Ulaanbaatar, Mongolia; 8 National Health Insurance Service, Jeonju, South Korea; 9 Society of Private Medical and Dental Practitioners, Accra, Ghana; 10 Ministry of Health, Ulaanbaatar, Mongolia; 11 Seoul National University College of Medicine, Seoul, South Korea; 12 Health Department of Arkhangai Province, Arkhangai, Mongolia; 13 Northwestern University Feinberg School of Medicine, Chicago, IL, 60611, USA

**Keywords:** Empanelment, panel maintenance, rostering, enrollment, continuity, population health management

## Abstract

Empanelment is a foundational strategy for building or improving primary health care systems and a critical pathway for achieving effective universal health coverage. However, there is little international guidance for defining empanelment or understanding how to implement empanelment systems in low- and middle-income countries. To fill this gap, a multi-country collaborative within the Joint Learning Network for Universal Health Coverage developed this empanelment overview, proposing a people-centered definition of empanelment that reflects the responsibility to proactively deliver primary care services to all individuals in a target population. This document, building on existing literature on empanelment and representing input from 10 countries, establishes standard concepts of empanelment and describes why and how empanelment is used. Finally, it identifies key domains that may influence effective empanelment and that must be considered in deciding how empanelment can be implemented. This document is designed to be a useful resource for health policymakers, planners and decision-makers in ministries of health, as well as front line providers of primary care service delivery who are working to ensure quality people-centered primary care to everyone everywhere.

## Disclaimer

The views expressed in this article are those of the author(s). Publication in Gates Open Research does not imply endorsement by the Gates Foundation.

## Introduction

An emerging body of evidence from across the globe shows that health systems based on a foundation of strong primary health care (PHC) are more efficient and produce higher value and better health outcomes ^1– 5^. As was recently reaffirmed at the Global Conference on Primary Health Care and through the Astana Declaration ^6^, PHC is a critical strategy for countries hoping to achieve the ambitious dual agendas recently adopted by the global health community: universal health coverage (UHC) ^7– 9^ and integrated people-centered health services (IPCHS) ^7, 10^.

A foundational strategy for building or improving PHC systems is empanelment (sometimes known as rostering in some parts of the world) ^11, 12^. Empanelment is the implementation of systematic, intentional, and continuously refined processes to identify and assign people to specific health care facilities, teams or primary care providers, which are then responsible for these people’s care. ^a^

In many health care systems, empanelment is an important early step towards effective and coordinated PHC and can begin a paradigm shift from disease treatment to disease prevention. Empanelment enables PHC systems to move from reactive care oriented around visits, to proactive care that leverages the primary health care team’s potential to improve population health.

By supporting health systems and providers to define and know the population to be served, empanelment can help deliver the right care at the right place and the right time. However, there is little international guidance for defining empanelment or understanding how to implement empanelment systems in low- and middle-income countries. This document aims to fill this gap by:

1. Proposing a people-centered definition of empanelment that reflects the responsibility to proactively deliver primary care services to all individuals in a target population.
2. Relaying a standard concept of empanelment regardless of how it is referred to within a country.
3. Describing why and how empanelment is used; and
4. Identifying key domains that may influence effective empanelment

The intended audience for this material includes health policy makers, planners and decision-makers in ministries of health, and those working at the front lines of service delivery.

## Empanelment: What is it?

In 2018, the Joint Learning Network for Universal Health Coverage (JLN) People-Centered Integrated Care collaborative, including participants from ten countries (China, Ghana, Indonesia, Malaysia, Mongolia, Morocco, South Korea, Sudan, Thailand and Vietnam) as well as a technical facilitation team from Ariadne Labs and Comagine Health, set out to identify a consensus definition of empanelment. We started by conducting a Google and PubMed search for existing definitions in use. Recognizing that there are multiple terms associated with or used in place of empanelment, we used the following search terms: “empanelment,” “empaneling,” “medical empanelment,” “patient registering,” “patient enrollment,” “attribution,” “patient assignment,” “rostering,” “patient rostering,” “panel management,” and “population health management.” Our search returned over 50 documents, with nine containing relevant definitions ^11– 19^. More details can be found in Appendix 2 of the JLN version of this document at http://www.jointlearningnetwork.org/resources/empanelment-a-foundational-component-of-primary-health-care.

However, the collaborative agreed that none of the nine definitions in use represented a definition that was clear, comprehensive, and actionable in their contexts. Therefore, building on the resources and recurrent review and input from the collaborative participants, the definition was iteratively improved until the following final definition was agreed to: ***Empanelment*** *(sometimes referred to as rostering)* ***is a continuous, iterative set of processes that identify and assign populations to facilities, care teams, or providers who have a responsibility to know their assigned population and to proactively deliver coordinated primary health care towards achieving universal health coverage.***

Empanelment should be people-centered and not provider-centered, based on the needs of the individuals served, and focused on ensuring that they feel as though they are treated as a whole person, not just as a set of diseases or conditions.

**Figure 1.**
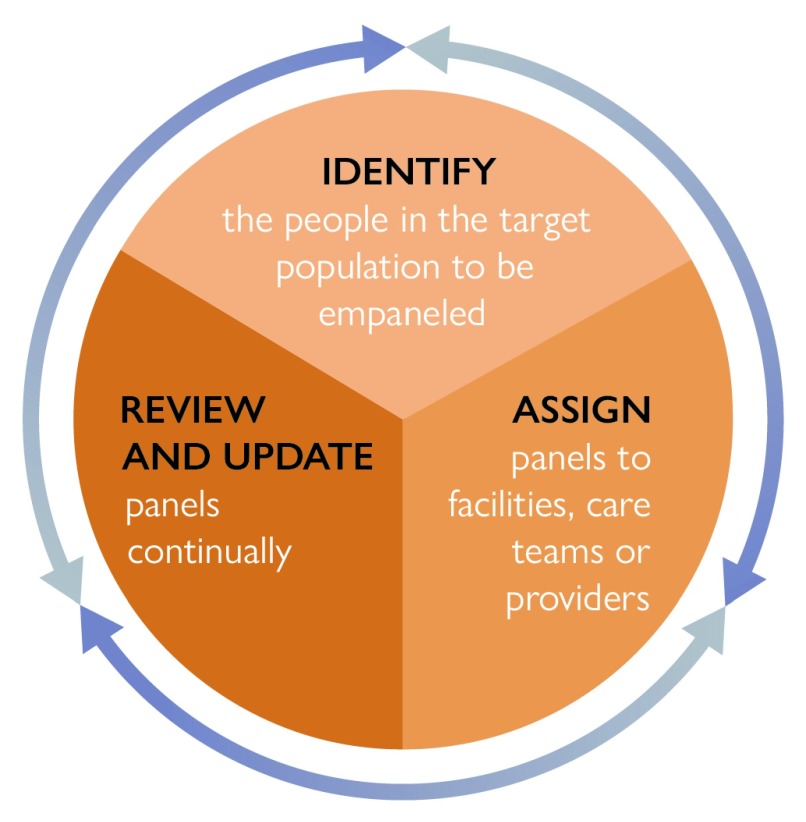
Phases and steps that support and enable effective empanelment.

Within the definition above are several phases and steps that support and enable effective empanelment, which are illustrated in Figure 1. 

## Empanelment: How to do it?

### PHASE 0: PREPARE

As pictured above, empanelment includes three key phases. However, before initiating implementation of these specific steps, there may be a need to complete some preparatory work. More specifically, the concept of empanelment and the inherent responsibility for the health and well-being of individuals in a panel may require a culture shift. Some potential targets to increase demand and readiness for empanelment include:

- Reframe and/or reinforce the **role of primary care** as the first point of contact with a responsibility to provide continuity, comprehensiveness, and coordination of care
- Provide empanelment **orientation and education** to all stakeholders, including the community and individuals
- Ensure a **trained and competent workforce** to deliver the primary care services inherent in accepting such responsibility
- Discuss the shift from **reactive to proactive health care** and the changes that may need to happen
- Assess and provide **training** about empanelment prior to full implementation (e.g. panel maintenance, population health management, creation of registries, etc.)
- Identify **local cultural contexts** that may impact how empanelment is implemented (e.g. women may prefer to see only female providers)
- Additionally, it is important to engage the community in empanelment, primarily by providing education and discussion about what empanelment is and what the benefits are to the individual and family in receiving care in this model.

The following sections provide additional details for the key phases needed to implement and sustain effective empanelment – 1) identify, 2) assign, and 3) actively review and update panel data. Note that there will be substantial diversity in the approaches to empanelment taken by different countries, reflecting different realities of resources, existing health system design and capacity, population health needs, and community input. This diversity leads to exciting and creative approaches to empanelment, implementation, and resulting primary care and population health management.

### PHASE 1: IDENTIFY

**Step one:** *Identify the target population for which the health care facility, team, or provider will be responsible.* Phase 1 requires identifying both the population of interest and the names of all individuals within this population. There are a variety of strategies, which may originate from geographic catchment areas or users of the facility. Additional strategies may include identifying individuals covered by a specific insurer or those identified by a ministry of health. A critical component of this process is to consider and identify individuals and vulnerable populations that might be missed when identifying the target population (e.g., individuals who do not have a permanent residence, sex workers, etc.)

**Step two:** *Once the entire target population is identified, develop or adapt a method to create a list of identifying characteristics (such as name, gender, and date of birth) for each member of that population.* In an optimized health system, all individuals in a population would be able to be identified and empaneled to a provider, team, or facility—described here as “full empanelment.” However, full and effective empanelment can be resource-intensive and may not be feasible in all settings or conditions. In these circumstances, ***selective empanelment*** can be used. This approach identifies sub-groups (e.g., individuals at high-risk or with chronic diseases such as diabetes or HIV) that may benefit from focused proactive tracking and management.

Countries often share several common barriers to the *Identify* phase, especially with the availability of data needed for empanelment. Specifically, in some countries or regions, the *number* of individuals is available, but the *names* of the individuals are not, nor is additional information included such as how to contact the individuals, making it impossible to implement effective empanelment. Additionally, it can be challenging to find effective methods to identify and track individuals in order to proactively provide care to an identified population ^13^. Ideally, an electronic health record or other technological solution can maintain the list of individuals. In some countries, all individuals are given an identification number at birth; these numbers are maintained in a database, which can be used to track health records. Other technology solutions might include a database of persons residing in a geographic catchment area or a health insurance database that contains information about providers and the people who have received care from those providers. If such databases are not available, other solutions such as spreadsheets or paper-based systems can be used as well. Individuals’ privacy and control over data exchange should be assured by complying with country laws and regulations that govern privacy.

### PHASE 2: ASSIGN

**Step three:** *From the list of identified individuals, assign a panel of individuals to each care team or provider.* A ***panel*** is a list of people assigned to a given health care facility, care team, or provider. People can be *empaneled* to a health care facility, identified care team, provider, or other entity. Approaches and algorithms for assigning individuals to panels can range from simple to complex and are often dictated by the ratios of providers to individuals. More complex approaches for assigning panels to specific providers or teams use risk stratification algorithms that account for acuity, equity, and care needs in targeting services toward particular groups. These algorithms may also incorporate factors such as age, sex, social needs, complexity of care needs based on clinical factors, previous health care costs accrued by individuals, and other factors.

Part of the process of assigning individuals may include making decisions about the size and composition of panels assigned to a provider, team, or facility. The simplest approach to these decisions involves assigning a specified number of individuals to a provider, team, or facility. Panel sizes vary widely and are based on many factors, including whether an optimized multidisciplinary team is in place. Different types of assignment approaches are illustrated in Table 1.

**Table 1.** Types of empanelment assignment approaches.

Below are approaches that are used to both **identify** the target population and to **assign** a panel of individuals to a facility, care team, or provider.		
**Geographic** This is the simplest approach and assigns people based on a clearly demarcated area where they live. Although geographic catchment areas may be used to define the boundaries of geographic assignment, the basic existence of catchment areas is not equivalent to geographic assignment.	**Insurance-based** In this approach, an insurer assigns individuals to a health care provider or team. The insurer may have a gatekeeping scheme in place through that particular assigned primary provider for referrals and access to other services. Individuals do not always adhere to insurance-based assignment, which can cause a mismatch between where the insurer assigns the individual and with whom (or where) care is received.	**Individual Choice** This approach, often referred to as voluntary, allows individuals to assign themselves by choosing their health care provider(s)/care team. With this approach, if an individual chooses a care team/provider, that individual will be added to the panel of the care team or provider. This approach runs the risk of missing individuals and families that do not seek care or who move often.

For example, in Mongolia, target panel sizes range from 1,500 to 1,800 for a team that includes one family medicine physician, four nurses, a pediatrician, and a senior midwife. However, in other countries and regions, panel sizes for a provider may range from 3,000 to 5,000—or higher ^20^. In some areas of Sudan, panel sizes can be as high as 10,000 due to the number of available providers. Ideally, the panel size should be balanced to ensure that the care team or provider can proactively deliver coordinated quality health care to all individuals in their panel. Note that panel size may affect the package of services that can be delivered.

**Step four:** *Conclude the assignment phase by ensuring that providers are aware of and acknowledge responsibility for their panels and individuals have been notified of their empanelment status.* It is critical that individuals be aware of and acknowledge the provider, team, or facility responsible for their care. This is known as “bilateral transparency” and/or “mutual association.” ^14^ Successful bilateral transparency means that people know who their team, provider, or facility is and the care team, provider, or facility knows the population for whom they are responsible, regardless of whether any particular individual from that population seeks care or not.

When multiple providers, care teams, or facilities are available and whenever possible, individuals’ preference for whom they would like to be empaneled to should be taken into account

### PHASE 3: ACTIVELY REVIEW AND UPDATE PANEL DATA

**Step five:** *Review and update panels on a regular basis.* Panels are not static; they are ever-changing and are affected by the life course of individuals. Panels change due to births, deaths, individuals relocating, changes in clinical status, and other life changes. Because of these possible changes, reviewing and updating on a regular basis is a key component of empanelment. The frequency of reviewing and updating panel data depends on local contexts and factors but is usually conducted annually or more frequently. The process of reviewing and updating panel data can be time-consuming and requires staff designated for panel maintenance.

***Panel maintenance*** involves a set of activities to ensure the delivery of primary health care services to all people and communities. Table 2 outlines important aspects of active panel maintenance.

**Table 2.** Aspects of active panel maintenance.

Activity	Rationale
**Determine panel size**	Ensures that a health care provider or team is assigned a reasonable number of people for whom they are responsible.
**Provide list of Individuals**	Enables providers and care teams to know and manage the individuals listed on their panel.
**Assess supply & demand**	Provides opportunities to balance the supply of providers and care teams with demand for services.
**Risk stratify**	Creates the ability to identify and optimally support high-risk individuals by providing enhanced services.
**Proactively manage**	Allows formation of registries to identify subgroups to ensure all evidence-based services are delivered.
**Optimize continuity**	Fosters continuity with the provider and care team as well as continuity across the care continuum.

In addition, in order for empanelment to drive improvements in health outcomes, the services delivered must be of sufficient quality to be effective and include promotive, preventive, curative, rehabilitative, and palliative health services, aligning with the WHO vision of universal health coverage. Merely producing a list of people based on residence in a catchment area and associating that list with a particular primary health care facility, team, or provider is unlikely to improve the delivery of PHC.

## Uses and benefits of empanelment: Why do it?

Empanelment is an important strategy that directly enables health systems to improve the patient experience, reduce costs and improve health outcomes. Empanelment supports primary health care systems to deliver on four key functions: first-contact accessibility, continuity, comprehensiveness, and coordination ^14^. Table 3 provides illustrative examples of the role of empanelment in each.

**Table 3.** Examples of the role of empanelment in the four key functions of primary health care.

Functions of PHC	Role of Empanelment
**First-contact** **access**	Empanelment is one of the primary enablers of making primary health care (PHC) the first point of contact for most people, most of the time. By establishing a system in which providers know the individuals for whom they are responsible, empanelment facilitates the delivery of more proactive outreach and care services. Similarly, within a system of empanelment, all individuals know who is responsible for their care and where they can go to receive services when needed. The establishment of panels also supports implementation of risk stratification systems which can be used to improve the accessibility of primary care services for those most in need ^21^.
**Continuous**	Empanelment establishes the basis for longitudinal relationships between individuals and their care provider/care team that can foster relational, managerial, and informational continuity over time ^22^. By designating a “home” for all people at the PHC level, empanelment facilitates both horizontal and vertical integration of care ensuring that individuals’ holistic care needs can be met at the PHC level and supporting informational continuity as people move between levels of care in the system.
**Coordinated**	Empanelment establishes the denominator of individuals for whom a specific facility/provider/care team is responsible and can be used to support the development and implementation of clinical pathways and dual referral systems. Patient panels provide a critical input for tracking patient movement through the health system to support patients during care transitions and improve coordination across different providers and levels of care.
**Comprehensive**	Empanelment—particularly to multidisciplinary care teams—supports the provision of proactive and comprehensive primary health care services.

## Conclusion

Effective empanelment requires assumption of responsibility for the health and well-being of a target population, including providing proactive primary health services based on each individual’s health status, which is a necessary step in moving towards people-centered integrated care.

This overview proposes a shared definition and language for empanelment with additional details on why and how empanelment is used. As countries grapple with higher demands on primary health care systems due to increasing incidence of non-communicable diseases, effective empanelment will be a critical component to optimize the management and health of target populations.

## Data availability

### Underlying data

No data are associated with this article

## Footnotes

*^a^ “Empanelment” is also known by a variety of other terms or in some countries, such as India, to refer to inclusion of hospitals as part of health insurance provider networks. This use of the term is unrelated to the process addressed in this document.*
