# Supplementary material for: Empanelment: A foundational component of primary health care
Source: Gates Open Res. 2019 Oct 31;3:1654. [Version 1] doi: 10.12688/gatesopenres.13059.1 (PMC7134391; doi:10.12688/gatesopenres.13059.1)
Supplement: Peer review history

# Peer review: Reviewer response for version 1

**DOI:** 10.21956/gatesopenres.14189.r28640

**Published:** 2020-04-03

**Authors:** Katherine Rouleau

**Affiliations:** 1 Department of Family and Community Medicine, University of Toronto, Toronto, ON, Canada

In this paper, the authors consider the concept of empanelment. Specifically, they propose a new definition for this process as well as five steps over three stages for its implementation. They also address the uses and benefits of empanelment.

 This is an excellent paper on an important and timely topic. The explicit framing of the concept of empanelment in the context of primary health care, the Declaration of Astana and the commitment to UHC at the onset imparts added value to the subject matter. The combination of a literature review and of a consensus process that drew perspectives from a range of settings to derive a conceptual definition of empanelment further strikes a fruitful balance between rigor, responsiveness and pertinence, leading to a definition of empanelment  that is, as suggested by the authors, “clear, comprehensive and actionable”.

 The article importantly highlights the link, albeit briefly, between empanelment, equity and quality. Furthermore, the description of the ways in which empanelment supports or relates to the four key characteristics of quality primary care services makes this paper particularly informative and useful for those wishing to take concrete steps to improve primary care services.

 The proposed phases and steps to empanelment are clear and helpful. The description of what is referred to a “phase 0”, the pre-conditions required to pave the way for empanelment, is particularly rich and useful as it clearly presents empanelment as one piece of a comprehensive approach to robust primary health care.

 The paper also paves the way for further reflection and possible debate on a number of points. First, rather than defining empanelment as a process that attaches patients to providers, teams or facilities, a subtle reframing as the assignment of providers, teams or facilities to a given group of patients might be a more patient-centered perspective of empanelment. This reframing highlights the accountability of the providers to the individuals and populations they serve, a step beyond the requirement for providers to “know” their patients as described in step 4 of the empanelment process. Second, while the paper provides a rich perspective on the advantages of empanelment in the care of individuals, it could have developed further the potential role of empanelment  in enabling health interventions at the population level and the integration of individual and population-based health intervention. This benefit of empanelment and its role in enabling the integration of individual and population-based measures might prove to be of particular interest to health planners and policy-makers in the post-Covid-19 era.

 In summary, this paper provides an intelligent, helpful and thorough presentation of the concept of empanelment and its potential contribution to PHC and UHC. The authors are commended for the quality of their work

I confirm that I have read this submission and believe that I have an appropriate level of expertise to confirm that it is of an acceptable scientific standard.

# Peer review: Reviewer response for version 1

**DOI:** 10.21956/gatesopenres.14189.r28157

**Published:** 2019-11-11

**Authors:** Kathryn A. Teng

**Affiliations:** 1 Department of Medicine, MetroHealth System, Cleveland, OH, USA; 2 Case Western Reserve University, Cleveland, OH, USA

In the Open Letter manuscript “Empanelment: A foundational component of primary health care,” the authors have attempted to provide international guidance for defining empanelment and how to implement empanelment systems in low- and middle- income countries.

 This effort is important because it provides a collaborative agreement as to the definition of empanelment and then provides phases and steps to support and enable effective empanelment. Many important concepts of empanelment are highlighted in this manuscript, including the role of primary care in providing continuous, comprehensive, and coordinated care. It is well known that effective primary care results in improved health outcomes and decreases overall healthcare spending.

 The step-phase approach to empanelment that is presented in this manuscript makes sense and provides good advice, as well as addresses potential challenges.

 The nuance to empanelment that I would have liked to see addressed more in the manuscript (or in a future manuscript) is the design of the care team. What does the right team look like to care for a specific patient panel? How does complexity of the patient panel or changes in individual complexity drive changes in the team structure? For example, how many nurse practitioners to physicians on a team? How many nurses on a team? How many social workers or behavioral health providers? In addition, how do social determinants of health factor into empanelment?

 Overall, this manuscript is timely, relevant, practical, and written for the correct target audience. I applaud the authors for the collaborative effort and discussion to improve primary care delivery around the world.

I confirm that I have read this submission and believe that I have an appropriate level of expertise to confirm that it is of an acceptable scientific standard.
